# Age-Related Macular Degeneration and Its Association With Neurodegenerative Disorders

**DOI:** 10.7759/cureus.34920

**Published:** 2023-02-13

**Authors:** Sankalp Mody, Abhishek Joshi

**Affiliations:** 1 Medicine, Jawaharlal Nehru Medical College, Datta Meghe Institute of Medical Sciences, Wardha, IND; 2 Community Medicine, Jawaharlal Nehru Medical College, Datta Meghe Institute of Medical Sciences, Wardha, IND

**Keywords:** retinal pigment epithelium (rpe), choroidal neovascularization, vascular endothelial growth factor (vegf), geographic atrophy, drusen

## Abstract

Age-related macular degeneration (AMD) is a highly prevalent macular condition that primarily affects the older population. It is the primary cause of blindness amongst the elderly population. It is an inflammatory disease that characteristically shows choroidal neovascularization and geographic atrophy. The exact pathomechanism of developing AMD is not known. However, certain factors such as increased age, smoking, genetic factors and certain environmental factors are usually associated with the development of the disease. AMD also involves oxidative stress-mediated destruction of retinal pigment epithelial cells and, consequently, that of retinal photoreceptors. Alzheimer's disease (AD) is a degenerative disorder involving the nervous system that usually affects people aged 65 and over. Both AMD and AD are age-related, degenerative conditions that have several similarities and share many of the same risk factors such as vascular conditions like arteriosclerosis, high blood pressure and obesity. It is believed that the early emergence of the clinical manifestations of AMD and AD may also be significantly influenced by oxidative stress and genetic polymorphism in complement factor H. A common pathogenic pathway between AD and AMD is quite likely. Amyloid-β is an aberrant protein that accumulates within the brains of Alzheimer's patients and appears as plaques on magnetic resonance imaging (MRI). These plaques are a pathognomonic sign of Alzheimer's disease. Similar to this, amyloid-β deposits are reported to build up beneath the retina of AMD patients, which appear as tiny clusters of protein-lipid substances known as drusen. It has also been found that individuals suffering from AMD exhibit an increased chance of developing AD than those with no AMD.

## Introduction and background

Age-related macular degeneration (AMD) is a chronic macular ailment that is progressive in nature and often affects those who are above the age of 75. It is characterized by impairment of macular vision owing to anomalies in the photoreceptor cells, retinal pigment epithelium (RPE) or the Bruch's membrane, which subsequently result in geographic atrophy and/or neovascularization [1]. In developed nations, AMD is the primary reason for permanent vision loss amongst the elderly population. It is the third-leading cause of blindness globally, just behind glaucoma and cataract. Nearly 200 million individuals worldwide suffer from AMD in some form [2]. Neovascular and non-neovascular are the two primary subtypes of AMD. Almost 80% to 85% of cases of AMD are of non-neovascular or dry type, and these typically have better visual prognoses. Neovascular or wet AMD accounts for the remaining 15% to 20% of the cases and contributes to about 80% of patients with blindness [3].

Apart from age, other significant etiological factors of AMD include cigarette smoking, an elevated body mass index, hypertension, hyperlipidaemia and heredity [4]. The Rotterdam Study showed that even after 20 years of quitting cigarettes, former smokers still have a higher chance of acquiring AMD [5]. It has been shown by various studies that hypertensive individuals are at an increased risk of developing AMD, which may be related to how systolic blood pressure affects blood flow within the choroid [6]. Additionally, hyperlipidaemia's significance as one of the primary reasons for AMD is a point of interest. According to some researchers, hypercholesterolemia may signal the beginning of geographic atrophy, whereas high levels of high-density lipoprotein (HDL) and fat consumption may be related to neovascular AMD [6]. Although the results of various studies on gender predisposition in AMD have been unconvincing, the incidence of AMD in females is relatively more [7].

Alzheimer's disease (AD) and age-related macular degeneration (AMD) have several similar characteristics, including the development of abnormal extracellular deposits (plaques and drusen), which are linked to neuronal degeneration [8]. Amyloid-β (Aβ) build-up in the extracellular space and hyperphosphorylated tau (p-tau) deposits within the cells are the hallmarks of AD. Aβ and p-tau deposits are accompanied by neuroinflammation and brain iron dyshomeostasis, which collectively cause slow neuronal death. Similarly, the build-up of iron and Aβ within the drusen and accumulation of p-tau and Aβ in the retinal ganglion cells (RGCs) along with inflammation imply an overlapping pathology between AMD and AD. In AD, visual abnormalities are common and are thought to arise before cognitive deterioration. Some are brought on by visual cortical degeneration, while others by RGC loss or AMD-related retinal degeneration [9].

## Review

Search methodology

**Figure 1:**
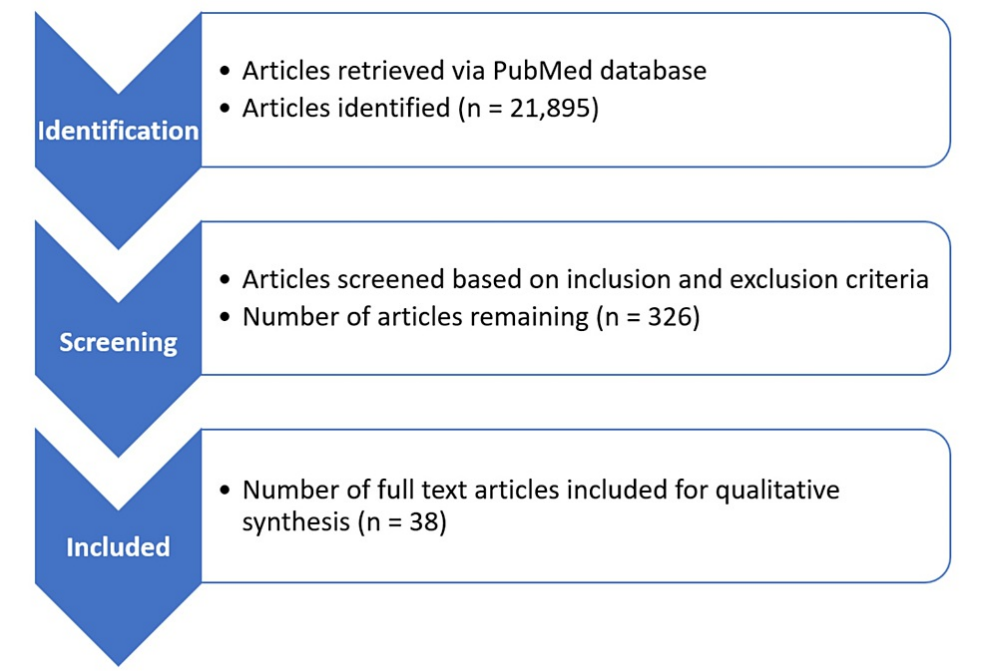
Search Methodology The figure is authors' own creation.

We searched PubMed databases for reviewing and shortlisting the relevant articles to accumulate evidence for our review article. The search strategy was tailored to individual databases and was as follows: ((((((Age related macular degeneration[Title / Abstract]) OR (AMD[Title / Abstract]) AND (“Alzheimer’s disease”[MeSH Terms]) AND (“Alzheimer’s”[MeSH Terms])))))). Furthermore, we screened the references list of potentially relevant studies to seek additional studies. Studies retrieved from these electronic searches and relevant references included in the bibliography of those studies were reviewed. We retrieved a total of 21,895 articles from the database. After screening by title, abstract and full-text availability, 326 articles remained. Amongst these, a total of 38 articles were finally included for the synthesis of evidence in this review article based on inclusion and exclusion criteria (Figure 1).

Pathophysiology

The retina, a transparent neural tissue layer that lines the inside of the eye's rear wall, is crucial for vision. The macula, which is the central region of the retina, gives humans their sharpest and most detailed image. The fovea, located in the macula's centre, is crucial for preserving the basic visual abilities required for tasks like identifying faces, driving vehicles and reading. AMD primarily impacts the outer retinal layers. These include RPE, Bruch’s membrane and choriocapillaris, along with underlying choroid. Bruch's membrane is crucial in forming neovascular lesions in AMD by mediating connections between the retinal pigment epithelium and choriocapillaris. AMD causes dysfunction and atrophy of the RPE, which also affects the photoreceptor layer of the retina and interferes with phototransduction. Such malfunction causes a breakdown in the signal pathway between the retina and the brain, which results in visual loss [10].

Additionally, microvascular insults that occur in conditions like hypertension and hyperlipidaemia are hypothesized to impact the choroidal vasculature. These microvascular injuries imply that the pathophysiology of the illness is influenced by both ischemic and inflammatory components [10]. In advanced neovascular AMD, abnormal angiogenesis, mediated by vascular endothelial growth factor (VEGF), is crucial for developing choroidal neovascular membranes (CNVs). For healthy choroidal and retinal vasculature, normal retinal circulation needs VEGF. However, hypoxia causes particular VEGF subtypes to express abnormally, resulting in the proliferation of new vascular channels which are vulnerable to bleeding and leaking that characterize neovascular AMD [11]. Numerous genes have also been implicated in the pathogenic mechanism of AMD [12]. The complement factor H gene was discovered as a primary culprit behind the development of AMD. It is also believed that drusen development is influenced by disruptions in complement-mediated regulatory function. The development of AMD is also linked to the non-complement mediated age-related maculopathy susceptibility 2 gene [13].

Ocular manifestations

The appearance of specific macular alterations, notably the accumulation of drusen, which are yellow-coloured extracellular localized deposits, is a pathognomonic sign of AMD. The likelihood of the illness progressing is influenced by the size and number of drusen. The diameter ranges for small, medium and giant drusen are 63 μm to 125 μm, respectively. Drusen may also be classified into two varieties: hard and soft. Hard drusen are often smaller and have more pronounced edges, whereas soft drusen can merge to produce larger, more dangerous lesions since their borders are less well-defined [14]. The Beaver Dam Eye Study revealed that individuals with larger soft drusen had nearly a 30% chance of developing advanced AMD [15].

**Figure 2:**
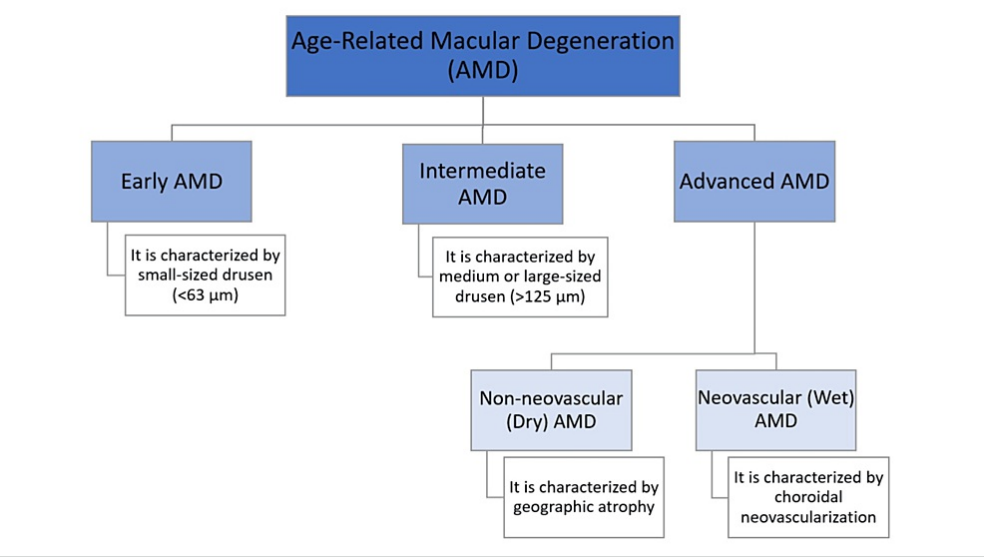
Classification of Age-Related Macular Degeneration The figure is authors' own creation.

AMD may also be classified into the following three types: early, intermediate and advanced neovascular AMD. Small macular drusen or a sparse cluster of medium-sized drusen have a lower propensity to progress to severe AMD and may not impair vision. Alterations in macular pigmentation may be a symptom of the initial stages of the disease, which also sets the stage for the advanced stage of the disease. A significant number of medium-sized drusen or only single large drusen, classified as intermediate AMD, carry a higher chance of developing into advanced illness and call for more careful monitoring [16]. According to the Age-Related Eye Disease Study (AREDS), age-related macular degeneration can be classified as shown in Figure 2.

Non-Neovascular (Dry) AMD

Amongst the initial signs of AMD is a drusen, which is clinically diagnosed as localized, pale yellow excrescences deep inside the retina, underneath the Bruch's membrane and RPE. Most drusen are hard or soft and range in size from 20 to 100 μm [17]. Hard drusen may be characterized as distinct, rounded yellowish spots. They are not influenced by ageing and are not associated with an increase in the incidence of neovascularization [18]. On the contrary, soft drusen are poorly defined with non-discrete boundaries, which measure 63 μm or more [19]. Clinically, geographic atrophy can be distinguished by a patch of the retina that is noticeably thinner than the surrounding retina and by a relative colour change that makes it easier to see the underlying choroidal capillaries. Good visual acuity may be retained if the foveal centre remains unharmed, yet reading vision may still be affected due to a restricted macular visual field [20].

Neovascular (Wet) AMD

A characteristic feature of wet AMD is neovascularization in the macula. The growth of new arteries from the choriocapillaris into the sub-pigment epithelial area following the tearing of Bruch's membrane is known as choroidal neovascularization (CNV) [21]. Within the last phase of the illness, neovascularization causes a disc-shaped fibrovascular scar around the macula, which causes long-term deterioration of the central vision [22]. Apart from the collection of fluid in the subretinal or intraretinal space and subretinal haemorrhage, a few other clinical indications of neovascular AMD are fat deposits, grey or greenish-yellow discolouration and detachment and rupture of the retinal pigment epithelium [23]. Haemorrhage, serous fluid collection or drusen accumulation under the retinal pigment epithelium can all result in a retinal pigment detachment (PED). A dome-shaped detachment of RPE, along with robust and diffuse hyperfluorescence and progressive pooling in a specific area, indicates serous PED. Haemorrhagic PED causes the RPE to darken because there is underlying blood, and all angiography phases show blocked fluorescence [24].

Association of AMD with Alzheimer’s disease

Several traits are shared between AMD-associated retinal degeneration and AD, such as extracellular Aβ deposits and oxidative stress, primarily mediated by iron accumulation. Apart from Aβ, the cerebral plaques seen in AD also consist of numerous lipid and protein components of drusen, such as vitronectin, apolipoprotein E, clusterin and components of complement activation, including C3 and C5b9. Additionally, the lesions of AMD, as well as AD, also show the presence of several metallic elements, including zinc, copper, iron and ubiquitin. Interestingly, in addition to the classical cerebral plaques found in the brains of AD patients, amyloid deposits have also been found within the inner layers of their retina [25].

Impairment of the functions of lysosomes and mitochondria plays a pivotal role in the development of AD and AMD. This results in a reduced capacity of the ageing cells of the brain and retina to clear the damaged cellular proteins leading to the formation of extracellular deposits. Uncontrolled activation of the complement system has also been implicated in the development of both diseases. It is believed that amyloid-β blocks the inactivation of C3b, resulting in uncontrolled activation of the complement system, which is responsible for the formation of drusen. This is proved by identifying various complement proteins in the drusen and the cerebral plaques in AMD and AD, respectively [26].

Mechanism of Retinal and Neuronal Degeneration

The retinal cells and a few other cells in the eye contain the type I transmembrane glycoprotein known as the amyloid precursor protein (APP) and other proteins implicated in AD. Two distinct post-translational processing pathways, amyloidogenic and non-amyloidogenic, are used in the brain to metabolize APP. The amyloidogenic pathway culminates in the generation of Aβ fragments following the breakdown of APP by the action of β-secretase and γ-secretase. The most harmful of them all, Aβ1−42, accumulates within the brains of Alzheimer's patients as extracellular plaques. Aβ aggregates bind reactive oxygen species such as metals, including iron and copper, resulting in mitochondrial damage and neurotoxic effects [27].

APP is metabolized similarly in the retina and other types of eye cells. Similar to the cerebrospinal fluid (CSF), pathogenic Aβ, soluble APPα and APPβ are all present in varying amounts dissolved within the aqueous as well as vitreous humour, and pathological Aβ deposits predominate in drusen in AMD. These findings are more pervasive and evident in AD mice models, which have proved to be essential in comprehending the contribution of Aβ in AD-related retinal degeneration. Several animal models of AD have demonstrated the presence of Aβ deposits and apoptotic RGCs [28].

It is not precisely known how the amyloidogenic pathway of APP breakdown in the retinal pigment epithelial (RPE) cells surpasses the non-amyloidogenic pathway. It is thought that when people age naturally, their RPE cells make and release more Aβ1-42, which collects within the intersection of these cells and the external part of the photoreceptor tips as well as in the area beneath the retina, followed by its microglial consumption [29]. This causes an inflammatory response that leads to the typical AMD drusen deposition. The role of Aβ in the pathogenesis of AMD is further supported by a recently conducted meta-analysis comprising 21 studies, which suggested a strong correlation between AD and AMD [30].

Nerve Growth Factor (NGF) as a Therapeutic Approach in AMD and AD

Nerve growth factor (NGF) is essential for the growth and survival of the neuronal cells of the peripheral and central nervous systems. The administration of NGF as a therapeutic agent in neurodegenerative disorders, especially AMD and AD, has shown promising results owing to its neuroprotective action on cerebral as well as extracerebral tissues such as the retina. When administered via the ocular route, NGF exerts a protective action on the RGCs and the photoreceptors and prevents their degeneration. It inhibits the degeneration of RGCs by decreasing the intracellular levels of pro-NGF and promoting the phosphorylation of tropomyosin receptor kinase A (TrkA). Various clinical trials have employed this anti-apoptotic effect of NGF to stop or prevent the further progression of visual loss. Following topical or intraocular administration of NGF, an increase in its systemic bioavailability is observed. This enhances the neuroprotective action of NGF on the eye-brain projections as well as on the nucleus basalis. Therefore, this approach can be employed to prevent neurodegenerative changes in the brain and the retina and slow down the progression of AD as well as AMD [31].

Diagnosis

Individuals above the age of 55 are advised to go for a routine fundus examination for early detection of macular degeneration. The presence of typical drusen, exudates, haemorrhage or geographic atrophy on fundus examination indicates AMD. Although the examination may account for most of the disease staging, using various imaging methods is now crucial for correlating examination findings and directing therapy.

Fluorescein Angiography

In the past, the gold standard for determining choroidal neovascularization in AMD had been fluorescein angiography (FA). Herein, fluorescein dye is introduced into the patient's vein, after which pictures of the chorioretinal circulation are recorded over several minutes. This invasive method may identify any exudation from various neovascular lesions [32].

Indocyanine Green Angiography

Patients with AMD were diagnosed and given treatment recommendations using indocyanine green angiography (ICG). This kind of angiography could distinguish the choroidal circulation more clearly than fluorescein angiography because of the properties of the dye. In individuals with dry AMD, indocyanine green angiography may detect plaques indicative of asymptomatic choroidal neovascularization, watershed zones indicative of potential future exudative transformation or regions of undetected CNV.

Optical Coherence Tomography

Optical coherence tomography (OCT) is a widely used technique that allows us to visualize the various layers of the retina in great detail. OCT may be compared to ultrasound, except that it uses light waves instead of sound waves to provide a complete cross-sectional picture showing all the layers of the retina along with the choroid. This makes it possible to recognize the specific layers of the retina that have been affected by AMD [33]. Optical coherence tomography angiography (OCT-A), a novel imaging technique, is based on optical coherence tomography, which improves the visibility of the complex choroid vascular network. This approach helps us understand the changes in neovascular AMD at the microvascular level when CNV lesions are present. This technique has largely taken the place of FA and ICG in these situations [34].

Treatment modalities

Laser Therapy

Until 2000, thermal laser therapy remained the basis of wet AMD treatment. By applying heat energy directly to the neovascular lesions, argon-laser photocoagulation causes CNV to regress with scar formation. It can induce CNV lesions to close when treated with a longer-wavelength infrared laser. Photodynamic treatment (PDT) was first introduced in 2000. In PDT, a photosensitive dye (verteporfin) is infused intravenously into the body. The body absorbs verteporfin, which builds up in CNV [35].

Intravitreal Injections and Anti-VEGF Agents

AMD treatment has been entirely changed by introducing localized intravitreal therapy combined with anti-VEGF therapy. At the level of pars plana, which is located 3 to 4 mm beyond the limbus, a tiny, 30-gauge needle can be used to safely and directly administer the required agent into the vitreous cavity. The manufacturer or compounding pharmacy dispenses the drug in a prefilled syringe, with the medication volume typically 0.05 mL [36]. Anti-VEGF medications effectively focus on CNV lesions and protect eyesight in wet AMD. Advanced AMD develops due to overexpression of VEGF, which causes leakage and neovascularization. One of the first mediators connected to the emergence of CNV was VEGF-A, which was also the initial focus of anti-VEGF therapy. Numerous VEGF-A isoforms exist, in addition to other angiogenic VEGFs such as placental-like growth factor (PLGF). The three commonly used intravitreal treatments for wet AMD are bevacizumab, ranibizumab and aflibercept [37].

Surgery

In the past, excision of neovascular lesions by surgery was considered a therapeutic choice; however; it has now mostly been abandoned in favour of more effective, minimally invasive alternative treatment options. The Sub-macular Surgery Trials, conducted in the 1990s, looked at how individuals with neovascular AMD fared after having sub-macular surgery for bleeding caused by CNV lesions. The study's findings indicated no advantage for those who received surgery, and the surgery arm saw a higher risk of problems.

## Conclusions

Age-related macular degeneration (AMD) is a major cause of blindness which primarily affects the older population. It induces detrimental changes within the deeper layers of the retina, along with the macula and the adjacent vasculature, thereby causing impairment of macular vision. Drusen, or retinal deposits, are a distinctive clinical trait of AMD. Based on certain specific characteristics, AMD can be classified into two broad types, neovascular (wet) and non-neovascular (dry). Dry or non-neovascular AMD is relatively more common than neovascular AMD. Neovascular AMD is characterized by the development of central choroidal neovascular membranes (CNVs) owing to aberrant vascular proliferation triggered by the action of vascular endothelial growth factor (VEGF). Therefore, intravitreally injected anti-VEGF is the chosen treatment for the neovascular type of AMD. AMD significantly impacts the quality of life of the senior population. The capacity to read, drive, identify people and carry out primary daily duties is affected by central vision loss. The phenotypic expression of the illness varies substantially. In the early form of the disease, the patient may experience little to no symptoms. On the contrary, in the later stages, the patient may complain of distortion of vision or even total loss of central vision. AMD is frequently linked to Alzheimer's disease (AD), which is considered to be the most common type of dementia among older people. In AD, there is an accumulation of amyloid-β (Aβ) in the extracellular space and hyperphosphorylated tau (p-tau) deposits within the cells, accompanied by neuroinflammation and brain iron dyshomeostasis. These changes collectively cause progressive neuronal death and dementia. Similarly, the build-up of Aβ and iron within the drusen in AMD suggests an overlapping pathogenic mechanism between the two diseases.
